# From Diagnosis to Complication: A Detailed Case Analysis on the Journey of Managing Medial Tentorial Dural Arteriovenous Fistula

**DOI:** 10.7759/cureus.70240

**Published:** 2024-09-26

**Authors:** Alali Meshari

**Affiliations:** 1 Department of Medical Specialties, College of Medicine, Majmaah University, Majmaah, SAU

**Keywords:** case study, complications, diagnosis, external carotid artery, medial tentorial dural arteriovenous fistula

## Abstract

Tentorial dural arteriovenous fistulas (DAVFs) are rare but highly dangerous vascular anomalies, constituting a small percentage of all intracranial DAVFs. Despite their infrequency, these lesions display aggressive characteristics, frequently leading to hemorrhage or neurological deficits due to their retrograde drainage into leptomeningeal veins, thus classifying them as Borden type III lesions.

This case presents a middle-aged man who suffered cerebellar and subarachnoid hemorrhages resulting from a medial tentorial DAVF. Initial imaging revealed a high-flow vascular lesion, which was subsequently confirmed through angiography. Endovascular embolization targeted the right middle meningeal artery, showing initial improvement. However, the patient experienced a notable decline two days later, attributed to residual or recurrent fistulas, venous hypertension, and cerebral edema.

Effective management of tentorial DAVFs necessitates a multidisciplinary approach, combining endovascular, surgical, and occasionally radiosurgical techniques. Continuous monitoring is essential for early detection and management of complications. This case underscores the critical need for a comprehensive strategy to manage the high risks associated with these vascular anomalies and to prevent potentially life-threatening outcomes.

## Introduction

Tentorial dural arteriovenous fistulas (DAVFs) are uncommon yet highly dangerous vascular lesions, representing only 4% to 8% of all intracranial DAVFs [1,2]. Despite their rarity, they are known for their aggressive clinical behavior, with a high incidence of hemorrhage or progressive neurological deficits occurring in 97% of cases [3] primarily due to their retrograde drainage into leptomeningeal veins, classifying them as Borden type III lesions [4].

Woimant et al. first reported a case of intracranial DAVF with spinal perimedullary venous drainage presenting as myelopathy in 1982 [5,6]. Since then, tentorial DAVFs have been increasingly recognized as a distinct and severe form of intracranial vascular anomalies. These lesions are most frequently diagnosed in middle-aged men, unlike other DAVFs, which are more common in older women [7-9]. The most common presenting symptom is spontaneous intracranial hemorrhage, typically caused by cortical venous reflux, where blood abnormally flows backward into the cortical veins [10]. Accurate diagnosis relies heavily on imaging techniques due to the nonspecific and nonlocalizing nature of the neurological symptoms associated with these lesions [11,12].

The treatment of tentorial DAVFs has significantly evolved over the years. While surgical resection was traditionally the primary treatment option, the deep-seated location of these lesions presents substantial challenges for surgical access [13-15]. Consequently, endovascular therapy has become the preferred first-line treatment, offering a less invasive approach with high success rates for fistula obliteration [16]. This method involves navigating the vascular system to deliver embolic agents that block abnormal blood flow. However, the extensive arterial supply from both the internal carotid artery (ICA) and vertebral artery complicates treatment, making the procedure technically challenging and increasing the risk of embolization compared to DAVFs fed by the external carotid artery (ECA) [17,18].

Previous cases of tentorial DAVFs typically present in middle-aged men and often result in spontaneous intracranial hemorrhages due to cortical venous reflux [7-8]. Despite their low incidence, these fistulas demand urgent medical attention because of their life-threatening nature [9,10].

In this report, we present a unique case that differs in its clinical presentation and management. To our knowledge, few cases have utilized a multidisciplinary treatment approach combining endovascular therapy and subsequent surgical intervention due to recurrent complications [11,12]. Our case contributes significantly to the literature by highlighting the critical role of vigilant management, aggressive follow-up, and timely intervention to prevent further complications [13,14].

Given the aggressive nature of tentorial DAVFs and their potential for severe complications, timely and effective management is crucial. A multidisciplinary approach, combining surgical, endovascular, and occasionally radiosurgical techniques, is often necessary to achieve complete occlusion and prevent recurrence. This case study emphasizes the importance of vigilant management for patients with medial tentorial dural AVFs to address the high risks associated with these vascular anomalies.

## Case presentation

**Figure 1:**
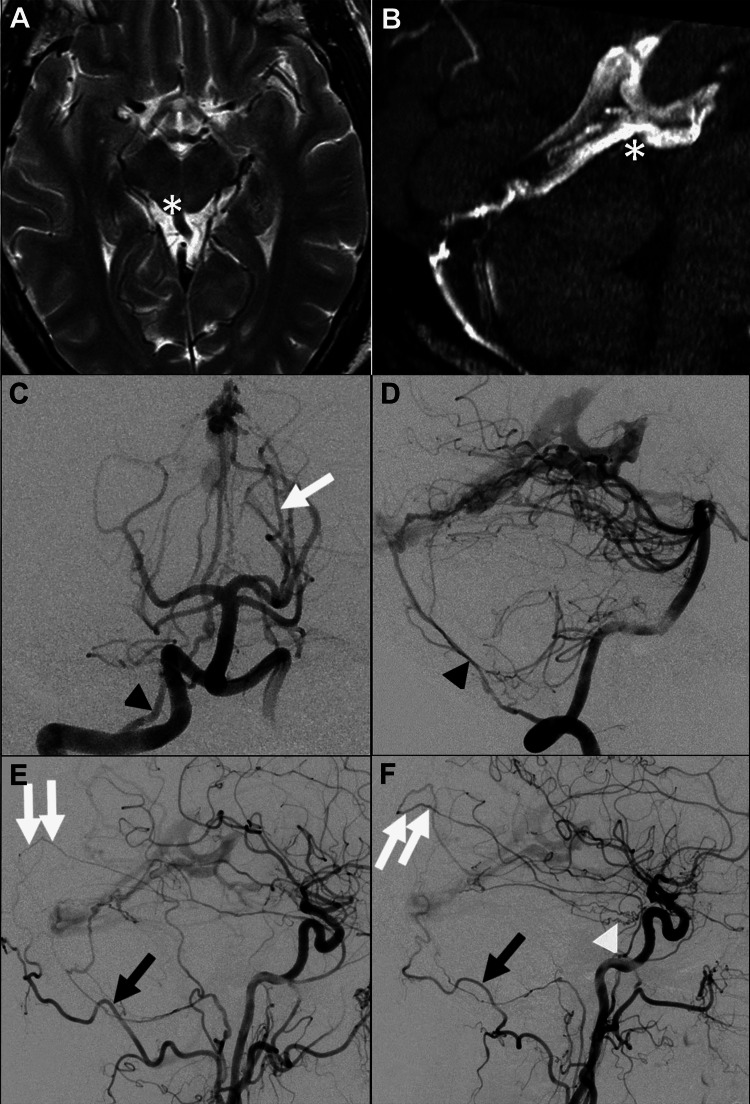
MRI and angiography of an enlarged cerebellar vein and deep vessels, with a tentorial dural arteriovenous fistula draining into the straight sinus. A) Axial T2-weighted MRI demonstrates a significantly enlarged cerebellar vein (indicated by a white asterisk) along with abnormally prominent deep vessels around the tentorium; B) sagittal maximum intensity projection MR angiography showing a significantly enlarged cerebellar vein (marked with a white asterisk); C) digital subtraction angiography (DSA) of the right vertebral artery, anteroposterior view, shows the tentorial dural arteriovenous fistula (DAVF) receiving blood supply from the right posterior meningeal artery (indicated by a black arrowhead); D) lateral view of the common carotid artery, indicating the left artery of Davidoff and Schechter (white arrow); E) lateral view of the left vertebral artery showing blood supply from the left and right occipital arteries (black arrows); F) digital subtraction angiography, anteroposterior and lateral views of the right external carotid artery, reveals the DAVF’s additional blood supply from the left and right middle meningeal arteries (represented by double white arrows), and tentorial branches from the right ICA (highlighted by a white arrowhead). The fistula drains into the straight sinus through the vein of Galen and retrograde outflow is observed in the superior and inferior sagittal sinuses and the deep venous system.

A middle-aged male patient was admitted to our facility following a sudden loss of consciousness at work. Initial imaging revealed a cerebellar hemorrhage and posterior circulation subarachnoid hemorrhage, suggesting a high-flow vascular lesion. The patient was referred to an otolaryngologist. Further angiographic studies confirmed the presence of a medial tentorial DAVF, characterized by an enlarged cerebellar vein and anomalous arteries on the tentorium (Figures 1A-1B). The fistula was associated with numerous source branches originating from the left and right occipital arteries, the left and right medial meningeal arteries (MMA), the right posterior meningeal artery, and the tentorial branches from the right ICA, and the left posterior cerebral artery (Figures 1C-1F). The fistula terminated in a dilated superior vermian vein, which then drained through the precentral cerebellar vein into the vein of Galen. Retrograde outflow was observed in the superior and inferior sagittal sinuses, as well as in the deep venous system, classified as a type III DAVF according to the Cognard scheme. The Davidoff and Schechter arteries were identified during angiography of the right vertebral artery (VA), as illustrated in Figure 1C.

**Figure 2:**
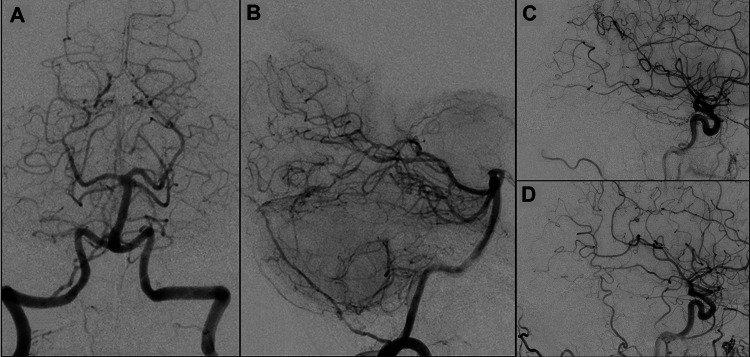
Post-treatment images showing complete occlusion of the tributary vessels to the fistula and normalization of the delayed venous phase. Post-treatment imaging in the anteroposterior (A) and lateral (B) views of the right vertebral artery, as well as the lateral view of the right common carotid artery in both arterial (C) and venous (D) phases, reveals complete occlusion of the tributary vessels associated with the fistula. Additionally, normalization of the delayed venous phase is observed.

The patient underwent embolization in two sessions due to the significant risks associated with a surgery requiring extensive brain exposure. A double femoral approach was implemented following the administration of general anesthesia and monitoring of systemic heparinization via activated clotting time. To access the fistula, a 6F Envoy catheter was inserted into the right ECA, and a Headway Duo microcatheter was navigated into the posterior-superior branch of the MMA using a Hybrid 12/14 guidewire (Balt, Montmorency, France). The DAVF was treated by injecting Onyx, a non-adhesive liquid embolic agent, under roadmap guidance. The embolization was performed using a slow and controlled injection technique, allowing the Onyx to fill the nidus of the fistula gradually. During the procedure, the injection was intermittently paused to let the Onyx solidify slightly before continuing, ensuring effective penetration and occlusion of the fistula and the proximal draining vein. The entire process took 32 minutes. During embolization, sequential DSA images were obtained from the right ECA. After the procedure, the tributary veins of the fistula were occluded entirely, while the straight sinus and the vein of Galen remained patent (Figure 2). The superior and inferior sagittal sinuses, as well as the deep venous system, regained their anterograde flow. The procedure was conducted without significant complications, and the patient initially showed improvement with complete tinnitus remission.

However, two days after the procedure, the patient experienced a significant decline, necessitating further investigation and intervention. Post-procedural computed tomography revealed rebleeding, correlating with the patient’s clinical deterioration, necessitating urgent surgical evacuation.

## Discussion

The management of tentorial DAVFs often presents significant challenges due to their complex nature and the potential for serious complications. Despite successful initial treatment, such as the embolization performed in this case, residual or recurrent fistulas can lead to notable clinical deterioration. Residual DAVFs may not be immediately detectable but can continue to cause symptoms due to abnormal blood flow. This emphasizes the need for thorough follow-up and possible additional interventions. Halbach et al. have highlighted that residual shunts can result in neurological decline, suggesting the importance of multiple treatment sessions and close monitoring [12]. Moreover, incomplete treatment may lead to the development of collateral vessels, which can sustain the risk of hemorrhage and neurological impairment [5].

Venous hypertension is another critical factor that can affect patient outcomes. This condition arises from impaired drainage of the fistula and can lead to increased venous pressure, contributing to cerebral edema and exacerbated hemorrhage. Awad et al. have reported that venous hypertension following DAVF treatment can significantly impair neurological function [4]. Miyachi et al. and Davies et al. further observed that persistent venous hypertension can cause worsened cerebral edema and recurrent hemorrhages, highlighting the necessity for effective hemodynamic management post-treatment [16,19].

Additionally, cerebral edema, which can result from the initial hemorrhage, the treatment process, or secondary venous hypertension, plays a crucial role in patient deterioration. Adamczyk et al. noted that DAVF recurrence is possible even after apparent successful embolization, with cerebral edema being a significant factor in neurological decline [20]. Managing intracranial pressure effectively is, therefore, essential to mitigate these risks.

In treating tentorial DAVFs, a multidisciplinary approach is crucial. Endovascular therapy is often the first-line treatment due to its minimally invasive nature and efficacy, but it must be carefully planned to address the complex arterial supply. If endovascular treatment proves challenging or incomplete, surgical intervention might be required to achieve complete occlusion. Radiosurgery can be a valuable adjunct, particularly for surgically inaccessible lesions. Ongoing monitoring and follow-up imaging are critical to detect and manage any recurrence or complications effectively.

## Conclusions

Tentorial DAVFs pose significant diagnostic and therapeutic challenges due to their aggressive clinical behavior and complex vascular anatomy. Effective management requires a tailored approach integrating endovascular, surgical, and radiological techniques. Continuous follow-up and monitoring are crucial for identifying and addressing potential complications early. This case underscores the critical role of a multidisciplinary team in successfully managing these high-risk vascular anomalies.

In conclusion, tentorial DAVFs are rare but highly dangerous lesions that demand prompt and effective treatment strategies to prevent life-threatening complications. A comprehensive, multidisciplinary approach - combining endovascular, surgical, and radiological techniques - is essential for successful outcomes. Continuous follow-up and monitoring are vital for detecting and managing any potential complications early, ensuring the best possible prognosis for the patient.
